# Genome-wide association study of mature cow size traits in American Angus cattle

**DOI:** 10.1007/s00335-026-10226-3

**Published:** 2026-04-06

**Authors:** Ayooluwa O. Ojo, Henrique A. Mulim, Milena A. F. Campos, André Garcia, Kelli Retallick-Riley, Pablo A. S. Fonseca, Hinayah R. Oliveira

**Affiliations:** 1 Purdue University, West Lafayette, IN USA; 2 Angus Genetics Inc. American Angus Association, St Joseph, MO USA

**Keywords:** Candidate gene, Energy balance, GWAS, Metabolism, Pleiotropic effect

## Abstract

Mature cow weight (MWT), height (MHT), and body condition score (BCS) are economically important traits that significantly influence cowherd profitability by affecting maintenance feed requirements, which is the largest component of production costs. This study aimed to identify genomic regions, candidate genes, and pleiotropic variants associated with mature cow size traits in American Angus cattle, and to characterize their related gene ontology terms and metabolic pathways. The dataset provided by the American Angus Association comprised 434,746 MWT records from 222,907 animals; 213,875 MHT records from 112,987 animals; and 382,156 BCS records from 209,696 animals. Of these, 45,606 cows were genotyped and imputed to a common marker density of 54,609 markers. A final dataset of 51,410 SNPs from 45,452 animals remained after quality control for further analyses. The single-step genome-wide association study (ssGWAS) method was used in the analyses. Significant associations for mature cow size were detected on BTA7, BTA14, and BTA20, overlapping with previously reported QTLs for growth, feed efficiency, and carcass traits. Candidate genes such as *FBXO32*, *STC2*, *DUSP1*, *CREBRF*, *LAP3*, and *PENK* were found to be related to muscle development, skeletal growth, lipid metabolism, and energy regulation. The genes *FBXO32*, *NTAQ1*, *ATAD2*, *DUSP1*, *ERGIC1*, *RPL26L1*, *ATP6V0E1*, *CREBRF*, *BNIP1*, *NKX2-5*, and *STC2* emerged as candidate genes exhibiting pleiotropic effects on mature cow size traits. Pathway enrichment highlighted the roles of insulin/IGF1R signaling, fibroblast growth factor receptors cascades, and mitogen-activated protein kinase pathways in MWT and MHT, while for BCS, only RHOD GTPase cycle was enriched. Pleiotropy-based analysis of regions affecting mature cow size traits identified shared genomic loci, and subsequent pathway analysis revealed G protein signaling as a common regulatory mechanism linking energy balance, adiposity, and growth. These results contribute to a better understanding of the genomic regions associated with mature cow size traits in Angus cattle.

**Supplementary Information:**

The online version contains supplementary material available at 10.1007/s00335-026-10226-3.

## Introduction

Beef cattle production plays a crucial role in global food systems, with efficiency and sustainability becoming increasingly important as demand for animal protein rises (FAO 2021). Among the traits that impact productivity and profitability, mature cow weight (MWT), height (MHT), and body condition score (BCS) are especially important because of their influence on cow maintenance costs, reproductive performance, and overall herd management (Northcutt et al. 1992; Ziegler et al. 2020; Kasimanickam et al. 2021; Zimmermann et al. 2021; Griffith And Rhinehart 2021). Early growth traits, such as weaning and yearling weights, have traditionally been the focus of genetic selection due to their direct link to meat production (Wang et al. 2020). However, mature cow size traits are a major driver of herd efficiency, reflecting the maintenance energy requirements and longevity (Freetly et al. 2023), and have recently become a focus of interest in breeding programs. Larger cows may wean heavier calves but also incur greater feed costs, making optimal mature size a critical balancing point in selection decisions (BIF, 2018). Thus, selection for increased growth without considering mature size may inadvertently increase maintenance costs (Herd And Oddy 2023). Although genetic correlations between mature cow size traits range from moderate to high (Ojo et al. 2025), the specific genes influencing mature cow size traits and the magnitude of their effects remain largely unknown. These traits are complex, shaped by both genetic and environmental factors, and their underlying genetic architecture is not yet fully understood.

Genome-wide association studies (GWAS) provide a powerful framework for identifying genomic regions and quantitative trait loci (QTL) associated with complex traits in cattle (Weller 2009). Beyond simply mapping loci, GWAS can reveal candidate genes and causal variants that offer insight into the biological pathways regulating traits of economic importance (Moser et al. 2010). Previous studies have identified QTL contributing to variation in body weight, conformation, and other structural traits in cattle (e.g., Kneeland et al. 2004; McClure et al. 2010; Santiago et al. 2017; Smith et al. 2022; Niu et al. 2025). For instance, Niu et al. (2025) reported candidate genes on BTA6 (*LAP3*,* MED28*,* NCAPG*, and *LCORL*) affecting body size in cattle. Smith et al. (2022) reported candidate genes on BTA6 (*LCORL*,* LOC782905*,* HERC6*,* SLIT2*, and *CCSER1*), BTA14 (*LOC112449660*), and BTA20 (*STC2* and *SH3PXD2B*) affecting growth traits in Angus cattle. Kneeland et al. (2004) also identified 40 haplotypes associated with birth weight, pre-weaning average daily gain, and post-weaning average daily gain on BTA2, BTA6, BTA14, BTA19, BTA21, and BTA23. Similarly, Bouwman et al. (2018) identified several genes influencing body size across multiple bovine breeds, including *PLAG1*, *NCAPG*, and *LCORL*. Importantly, some of these loci exhibit pleiotropic effects, where a single gene or genomic region influences multiple traits simultaneously (Utsunomiya et al. 2013; Seabury et al. 2017; Smith et al. 2019; Zhang et al. 2020). Such pleiotropic QTL can be detected through multi-trait GWAS meta-analysis (Bolormaa et al. 2014), highlighting the value of integrating multiple analytical approaches to uncover shared biological mechanisms.

A single-trait GWAS can identify loci specific to one trait, but pleiotropic variants that influence multiple size-related traits may be overlooked if traits are analyzed independently. Multi-trait and pleiotropy-based approaches, such as the meta-analysis described by Bolormaa et al. (2014), improve power to detect shared loci, reduce false negatives, and provide insight into the biological pathways that jointly regulate multiple traits. Combining these complementary approaches allows for the distinction between trait-specific loci and pleiotropic loci, which has direct implications for balancing productivity and efficiency in breeding programs. Recent studies that used this approach have identified pleiotropic loci across diverse traits and species. For example, Niu et al. (2025) reported candidate genes with pleiotropic effects on body size in beef cattle using multi-omics data. Yao et al. (2024) used a pleiotropy-based approach to identify genes with pleiotropic effects on fat deposition traits in cattle. Bouwman et al. (2018), through a meta-analysis of GWAS for bovine stature, identified common genes, including *PLAG1*, *NCAPG*, *LCORL*, and *CHCHD7*, that influence body size across mammalian species. Similarly, Oliveira et al. (2024) identified pleiotropic variants associated with physiological and anatomical indicators of heat stress response in lactating sows. However, to the best of our knowledge, no study has applied pleiotropy-based approaches specifically to mature cow size traits.

The American Angus Association (AAA) maintains one of the largest and most comprehensive beef cattle datasets globally, combining high-density genomic information with detailed phenotypic records on MWT, MHT, and BCS. This resource provides an exceptional opportunity to identify loci with small-to-moderate effects and to gain deeper insight into the genetic and biological mechanisms underlying variation in mature cow size (Moser et al. 2010). Using this dataset, the objectives of the present study were to: (1) identify genomic regions, candidate genes, and pleiotropic variants associated with mature cow size traits (i.e., MWT, MHT, BCS) in American Angus cattle; and (2) characterize the biological processes underlying these traits and prioritize their potential functional candidate genes.

## Materials and methods

Phenotypic records on MWT, MHT, BCS, pedigree, and genomic information were obtained from the American Angus Association database. Therefore, approval from the Animal Care and Use Committee was not required for this study. The datasets and quality control procedures for the phenotypic records followed those described in our previous study (Ojo et al. 2025). In summary, quality control excluded records from cows younger than one year or older than 15 years due to limited data at these ages. Contemporary groups (CG) were defined by herd, year, and season at which mature cow trait measurements were taken. The CGs with fewer than three animals were excluded. For MWT and MHT, outlier records more than three standard deviations from the CG mean were removed, whereas for BCS, CGs without variability were removed. After quality control, the final dataset included 434,746 MWT records from 222,907 cows, 213,875 MHT records from 112,987 cows, and 382,156 BCS records from 209,696 cows.

### Genotypic information

A total of 45,606 cows were genotyped and imputed to a common marker density of 54,609 SNPs. Imputation was performed by Angus Genetics Inc. using the FImpute software (Sargolzaei et al. 2014) as part of their official genomic evaluation pipeline. Among all genotyped animals used in this study, 33,764 had phenotypic records, while 11,842 animals were genotyped but did not have their own phenotypes. Genomic quality control (QC) was performed using the preGSf90 program (Aguilar et al. 2014). Animals and SNPs with a call rate < 0.90, SNPs located on non-autosomal chromosomes, SNPs with unknown or duplicated position, and SNPs with minor allele frequency (MAF) < 0.05 were removed from subsequent analyses. SNPs showing an absolute difference > 0.15 (p-value ≥ 1 × 10^–6^) between observed and expected heterozygosity under Hardy–Weinberg equilibrium, as described by Wiggans et al. (2009), were also removed, as such deviations may indicate potential genotyping errors. After quality control, 51,410 SNPs and 45,205 animals remained for further analyses.

### Single-step genome-wide association studies for mature cow size traits

Single-step genome-wide association study (ssGWAS) was performed using the BLUPF90 + family of programs (Misztal et al. 2002, 2020; Lourenco et al. 2020), following the approach described by Wang et al. (2012b). Mixed-model equations were solved using the following single-trait repeatability model:

1 $$\:\mathbf{y}\:=\:\mathbf{X}\mathbf{b}+\mathbf{Z}\mathbf{a}+\mathbf{W}\mathbf{p}\mathbf{e}+\mathbf{e}$$

where **y** is the vector of phenotypic observations recorded in the cows; **b** is the vector of fixed effects related to the contemporary group (farm, year and season of measurement), parity, and age in days fitted as a linear covariate nested within age in years; **a** is the vector of random additive genetic effects; **pe** is the vector of random permanent environmental effects; and **e** is the vector of random residual effects. The **X**,** Z**, and **W** are the incidence matrices associating **b**,** a**, and **pe** to the phenotypic observations, respectively. The variances of **a**,** pe**, and **e** are represented as follows:

2 $$\:\mathrm{V}ar\left[\begin{array}{c}\boldsymbol{a}\\\:\boldsymbol{p}\boldsymbol{e}\\\:\boldsymbol{e}\end{array}\right]=\:\left[\begin{array}{ccc}\mathbf{H}{{\upsigma\:}}_{\mathrm{a}}^{2}&\:0&\:0\\\:0&\:\mathbf{I}{{\upsigma\:}}_{\mathrm{p}\mathrm{e}}^{2}&\:0\\\:0&\:0&\:\mathbf{I}{{\upsigma\:}}_{\mathrm{e}}^{2}\end{array}\right]$$

where $\:{{\upsigma\:}}_{\mathrm{a}}^{2}$ is the direct additive genetic variance for each trait; $\:{{\upsigma\:}}_{\mathrm{p}\mathrm{e}}^{2}$ is the permanent environmental variance; and $\:{{\upsigma\:}}_{\mathrm{e}}^{2}\:$is the residual variance; **H** is the matrix that combines pedigree and genomic information (Aguilar 2010); and **I** is an identity matrix. Variance components ($\:{{\upsigma\:}}_{\mathrm{a}}^{2}$, $\:{{\upsigma\:}}_{\mathrm{p}\mathrm{e}}^{2}$, and $\:{{\upsigma\:}}_{\mathrm{e}}^{2}$) were fixed to the pedigree-based estimates reported in Ojo et al. (2025). The inverse of the **H** matrix was computed as:

3 $$\:{\mathbf{H}}^{-1}={\mathbf{A}}^{-1}+\:\left[\begin{array}{cc}0&\:0\\\:0&\:{\mathbf{G}}^{-1}-{\mathbf{A}}_{22}^{-1}\end{array}\right]$$

where **A** is the relationship matrix based on the pedigree information for all animals; **A22** is the pedigree-based relationship matrix for genotyped animals; and **G** is the genomic relationship as described in the first method proposed by VanRaden (2008):

4 $$\:\mathbf{G}=\frac{\boldsymbol{Z}{\boldsymbol{Z}}^{\boldsymbol{{\prime\:}}}}{2\sum\:{\mathrm{p}}_{\mathrm{i}}(1-{\mathrm{p}}_{\mathrm{i}})}$$

where **Z** is a centered incidence matrix of genotype covariates (0, 1, 2) and $\:2\sum\:{\mathrm{p}}_{\mathrm{i}}(1-{\mathrm{p}}_{\mathrm{i}})$ is a scaling parameter, where $\:{p}_{i}$​ is the frequency of the reference allele at the *i*th SNP. In ssGWAS, SNP effects were derived by back-solving from genomic estimated breeding values (GEBVs). First, breeding values for genotyped animals were expressed as (Misztal et al. 2020):

5 $$\:\mathbf{a}=\mathbf{Z}\mathbf{u}$$

where **a** is the vector of GEBVs for genotyped individuals calculated using BLUPF90+; **Z** is the incidence matrix relating individuals to SNP marker effects; and **u** is the vector of SNP substitution effects. The SNP effects were then estimated as:

6 $$\:\widehat{\mathbf{u}}={\mathbf{Z}}^{\mathbf{{\prime\:}}}{\left(\mathbf{Z}\mathbf{I}{\mathbf{Z}}^{\mathbf{{\prime\:}}}\right)}^{-1}\widehat{\mathbf{a}}$$

where **û** is a vector of estimated SNP effects; **I** is an identity matrix; **Z** is the incidence matrix relating individuals to SNP marker effects, and $\:\widehat{\mathbf{a}}$ is the vector of GEBVs. The SNP effects were assumed to follow the infinitesimal model, with equal variance across loci. Given the genetic homogeneity characteristic of the Angus breed (Mulim et al. 2025), which is consistent with the results from a principal components analysis performed in our dataset (Supplementary Material S1), principal components were not added in the statistical models due to the absence of evidence for population stratification.

The p-values for each SNP were obtained with the postGSF90 program (Aguilar et al. 2019) as:

7 $$\:{\mathrm{p}}_{i}=2(1-{\Phi\:}(\left|\frac{{\alpha\:}_{i}}{SD\left({\alpha\:}_{I}\right)}\right|\left)\right)$$

where αi is the SNP effect estimate; SD is the standard deviation; and Φ is the standard normal cumulative distribution function.

To control for multiple testing, a Bonferroni correction (alpha = 0.05) was applied based on the number of independent chromosomal segments (Corbin et al. 2012). The number of segments was estimated using the average chromosome length, and the effective population size (Ne = 182; Lozada-Soto et al. 2020), providing chromosome-wide significance thresholds (Goddard et al. 2011).

### Pleiotropic effects

Bolormaa et al. (2014) proposed a GWAS meta-analysis that allows the detection of pleiotropic variants across groups of more than two traits. This method combines the results from single-trait GWAS to compute a unified multi-trait test statistic. Notably, it has been shown to achieve a lower false discovery rate (FDR) compared to single-trait analyses at the same significance threshold (Bolormaa et al. 2014, 2016; Xiang et al. 2017; de Oliveira et al. 2024). In summary, phenotypes are first pre-adjusted for fixed effects, and for animals with repeated records, the average of the adjusted phenotypes was used as the response variable in the GWAS, and the resulting GWAS outputs were used in the multi-trait association analysis. To meet the assumption of trait independence, originally intended for meta-analyses across independent studies, the phenotypes were subsequently decorrelated using Cholesky transformation (Bolormaa et al. 2014; Xiang et al. 2017, 2020).

The Cholesky transformed traits (CTs) were calculated based on centered, and z-score-scaled mature cow size traits in R (v4.4.0). Given *n* number of animals and *k* number of mature cow size traits, an *n × k* (number of traits = 3) CT matrix was calculated based on Cholesky decomposition:

8 $$\:{c}_{n}={L}^{-1}{g}_{n}$$

where, $\:{c}_{n}$ is a *k ×* 1 vector of Cholesky scores for the animal *n*; *L* is the *k × k* matrix of the Cholesky factor satisfying $\:L{L}^{t}=COV$, the *k × k* covariance matrix of raw mature cow size traits after standardization as z-scores; and $\:{g}_{n}$ is an *k ×* 1 vector of raw mature cow size traits for animal *n.* The *k*th CT can be interpreted as the *k*th raw mature cow size trait corrected for the preceding $\:k\:-\:1$ trait(s), while the first CT equals the first raw trait. The Cholesky transformation was applied in the order of the traits: BCS, MWT, and MHT. Consequently, the first CT corresponds to BCS, the second (MWT_CT_) represents mature cow weight corrected for BCS, and the third (MHT_CT_) reflects mature cow height corrected for both BCS and MWT. This orthogonalization removes shared variance between traits in the specified order, enabling genome-wide analysis by modeling the chi-square ($\:{\chi\:}^{2}$) distribution of SNP effect sizes. The final CT used in the analyses included 209,614 BCS_CT_ records, 209,507 MWT_CT_ records, and 108,633 MHT_CT_ records, with a single record per animal.

Multi-trait association analysis of the three CTs was performed following previously described procedures (Bolormaa et al. 2014, 2016). Briefly, the multi-trait $\:{\chi\:}^{2}$ statistic for the *i*th SNP was calculated based on its signed t-values generated from each single trait GWAS of the CTs:

9 $$Multi - trait \:{\chi\:}^{2}={\boldsymbol{t}}_{\boldsymbol{i}}^{\boldsymbol{{\prime\:}}}{\boldsymbol{V}}^{-1}{\boldsymbol{t}}_{\boldsymbol{i}}$$

where $\:{\boldsymbol{t}}_{\boldsymbol{i}}$ is a k (number of traits = 3) *×* 1 vector of the signed t-values (i.e., effect/SE) of SNP i for the k traits; $\:{\boldsymbol{t}}_{\boldsymbol{i}}^{\boldsymbol{{\prime\:}}}$ is a transpose of vector $\:{t}_{i}$ (1*×* 3); and $\:{\boldsymbol{V}}^{-1}$ is an inverse of the *k × k* correlation matrix, where the correlation between two traits is the correlation over the 51,410 estimated SNP effects (signed t-values) of the two traits. The $\:{\chi\:}^{2}$ value of each SNP was tested for significance based on a $\:{\chi\:}^{2}$ distribution with *k* degrees of freedom, where the null hypothesis declared that the SNP had no significant effects on any one of the *k* traits. A Bonferroni correction was also applied, as previously described for the single-trait ssGWAS.

### Gene annotation, QTL identification and functional analyses

Gene and QTL annotation were performed using the GALLO package(Fonseca et al. 2020) available in the R software v.4.4.0 (R Core Team 2024). The gtf file used for gene annotation corresponding to the ARS-UCD1.2 of the bovine genome was obtained from Ensemble database. For the QTL annotation, the gff file from Cattle QTLdb corresponding to the ARS-UCD1.2 of the bovine genome was used (Rosen et al. 2020). A genomic window spanning 100 kb upstream and downstream of each significant SNP was used to identify nearby QTLs and genes, based on the extent of linkage disequilibrium (LD) decay observed in the population (Supplementary Material S2). Annotated regions were then compared against previously reported cattle QTLs from the Animal QTL Database(Hu et al. 2013) to identify overlaps and assess potential biological relevance. For biological interpretation, QTLs were classified into six categories: Production, Meat and Carcass, Health, Milk, Exterior, and Reproduction. Enrichment analysis was performed to test for over-representation of the identified QTLs within these major trait categories. The GALLO package was also used to perform a QTL enrichment analysis for each annotated trait category by chromosome. The enriched QTL were defined based on a false-discovery rate (FDR) < 0.05. Functional enrichment analyses were performed using the gprofiler2R package (Kolberg et al. 2020), including Gene Ontology categories (biological process, molecular function, and cellular component) and curated pathway databases such as Kyoto Encyclopedia of Genes and Genomes (KEGG), Reactome and WikiPathways.

### Comparative analysis of potential candidate genes with a meta-analysis

The meta-analysis by Bouwman et al. (2018) investigated the genetic architecture of stature across 17 cattle populations using over 25 million imputed whole-genome sequence variants. Their findings revealed that stature in cattle, similar to humans, is highly polygenic, with lead variants in 163 genomic regions explaining up to 13.8% of the phenotypic variance. Notably, there was substantial overlap in stature-associated loci among cattle, humans, and dogs, suggesting the involvement of a conserved set of genes regulating body size across mammals. To enable direct comparison between the results of our study and those from Bouwman et al. (2018), we first performed a genome coordinate conversion (liftover). The liftover process was conducted using the rtracklayer package in R (v.4.4.0) and the UCSC chain file bosTau8ToBosTau9.over.chain, which aligns the *Bos taurus* UMD3.1 assembly to the ARS-UCD1.2 reference genome (Perez et al. 2025). Following coordinate alignment, gene and QTL annotations were performed as previously described, using the corresponding gtf and gff files. Positional candidate genes were identified within a ± 500 kb window upstream and downstream of each SNP. The list of annotated genes from our study was compared with the meta-analysis study, and shared genes between the two datasets were identified. The relationship between these shared candidate genes and enriched QTLs was investigated using the NetVis function in GALLO, where nodes represented genes and QTLs, and edges indicated shared genomic associations.

## Results

### Genome-wide association studies for mature cow size traits

**Fig. 1:**
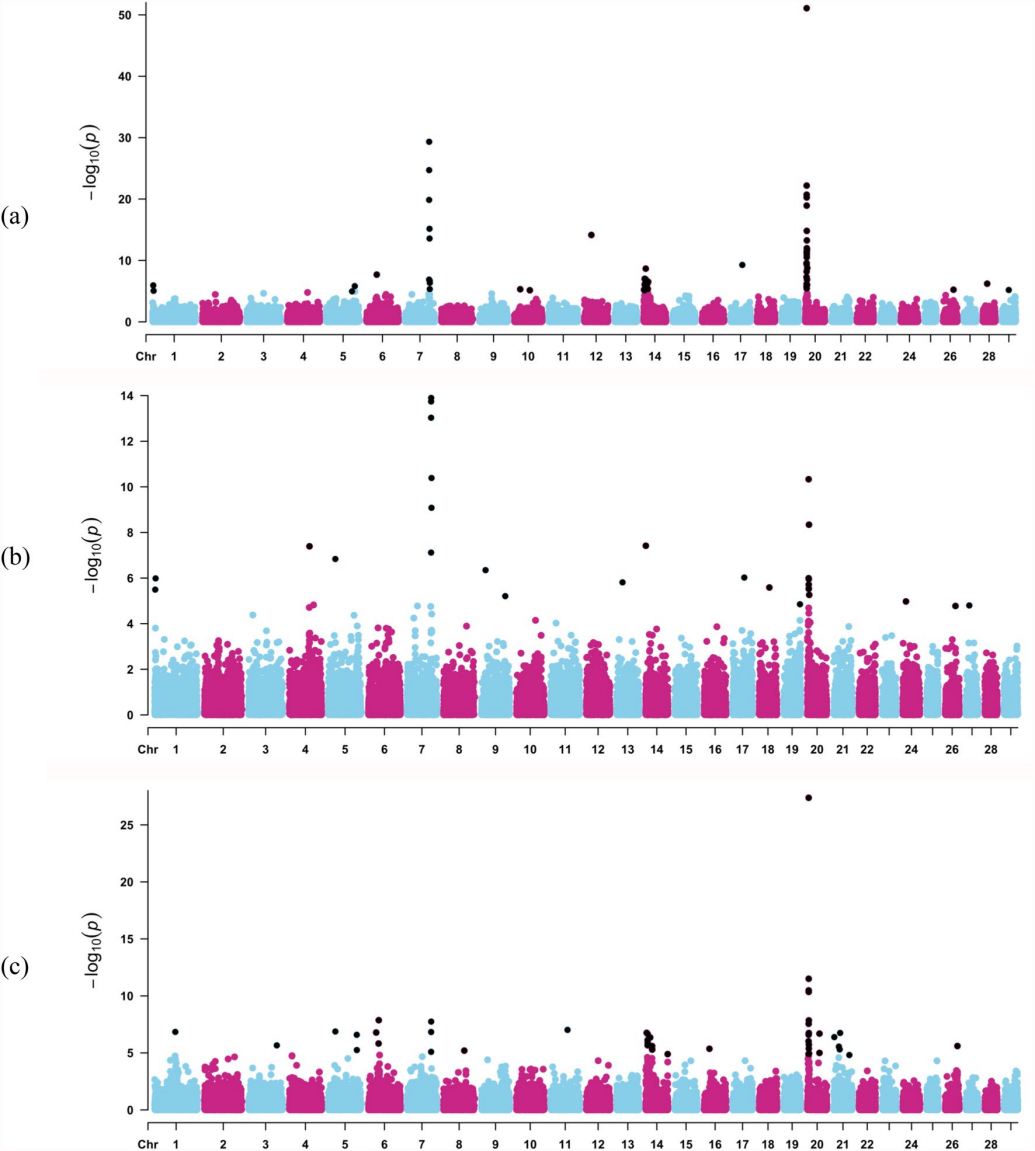
Manhattan plot for **a** mature cow weight, MWT; **b **mature cow height, MHT; and **c **body condition score, BCS, in American Angus cattle population. Black dots represent the significant markers

The estimated number of independent chromosomal segments (Me) per chromosome and the corresponding adjusted chromosome-wide significance thresholds are reported in the Supplementary Tables S3. Figure 1a shows the Manhattan plot for MWT in the American Angus Cattle population. A total of 59 significant SNP markers associated with MWT were identified. These markers were distributed across twelve chromosomes (i.e., BTA1, BTA5, BTA6, BTA7, BTA10, BTA12, BTA14, BTA17, BTA20, BTA26, BTA28, BTA29). The significant SNPs explained 6.26% of the genetic variance of MWT, with those on BTA20, BTA7, and BTA14 accounting for 3.85%, 1.38% and 0.83%, respectively. In total, 122 genes were identified in these regions, of which 68 were protein-coding, 47 long non-coding RNAs, 6 microRNAs, and 1 ribosomal RNA. Some of the genes unique to MWT include *ANK3*, *ATOSA*, *CCDC166*, *EPPK1*, *FAM83H*, *GFUS*, *HAUS4*, *IQANK1*, *LRP10*, *MAB21L1*, *MAPK15*, *MMP14*, *MRPL52*, *MSX2*, *NBEA*, *NSMCE2*, and *ZFAT*.

Figure 1b shows the Manhattan plot for MHT. Forty-seven markers located on 12 chromosomes (BTA1, BTA3, BTA5, BTA6, BTA7, BTA8, BTA11, BTA14, BTA16, BTA20, BTA21, and BTA26) were shown to be associated with MHT. Together, these regions explained 3.07% of the total additive genetic variance for MHT, with those on BTA20, BTA14, and BTA7 accounting for 2%, 0.63% and 0.37% respectively. These markers overlapped with 111 genes, being 67 protein-coding, 35 long-noncoding RNAs, 5 microRNAs, 1 ribosomal RNA, and 3 small nucleolar RNAs. Genes exclusively associated with MHT include *ADAMTSL3*, *AEN*, *ALPK3*, *ASB2*, *ATP10A*, *BCCIP*, *BPNT2*, *CCDC197*, *DEPTOR*, *DHX32*, *EDRF1*, *FAM181A*, *FAM184B*, *FANK1*, *GKAP1*, *HNRNPK*, *ISG20*, *KIF27*, *LAP3*, *MDH1*, *MED28*, *PDE8A*, *PENK*, *QNG1*, *SDR16C6*, *SLC28A1*, *TP63*, *UROS*, *USH2A*, and *WDPCP*.

The Manhattan plot for BCS is shown in Fig. 1c. A total of 29 significant SNP markers were statistically associated with BCS. Relevant genomic regions were located on BTA1, BTA4, BTA5, BTA7, BTA9, BTA13, BTA14, BTA17, BTA18, BTA19, BTA20, BTA24, BTA26, and BTA27. The significant SNPs explained 1.75% of the total additive genetic variance for BCS, where significant SNPs on BTA20, BTA7, and BTA4 explained 0.77%, 0.76%, and 0.09%, respectively. The significant markers overlapped with 82 genes, which have been classified as protein-coding (42), long noncoding RNAs (36), microRNAs (2), ribosomal RNA (1), and small nucleolar RNA (1). Some of the genes unique to BCS include *AKAP12*, ARHGAP39, *C14H8orf33*, *C14H8orf82*, *COMMD5*, *FILIP1*, *GPT*, *LRRC14*, *LRRC24*, *MFSD3*, *MTHFD1L*, *NEBL*, *PPP1R16A*, *RECQL4*, *RPL8*, *TMEM30A*, *ZFHX3*, *ZNF16*, *ZNF250*, *ZNF34*, and *ZNF7*.

**Fig. 2:**
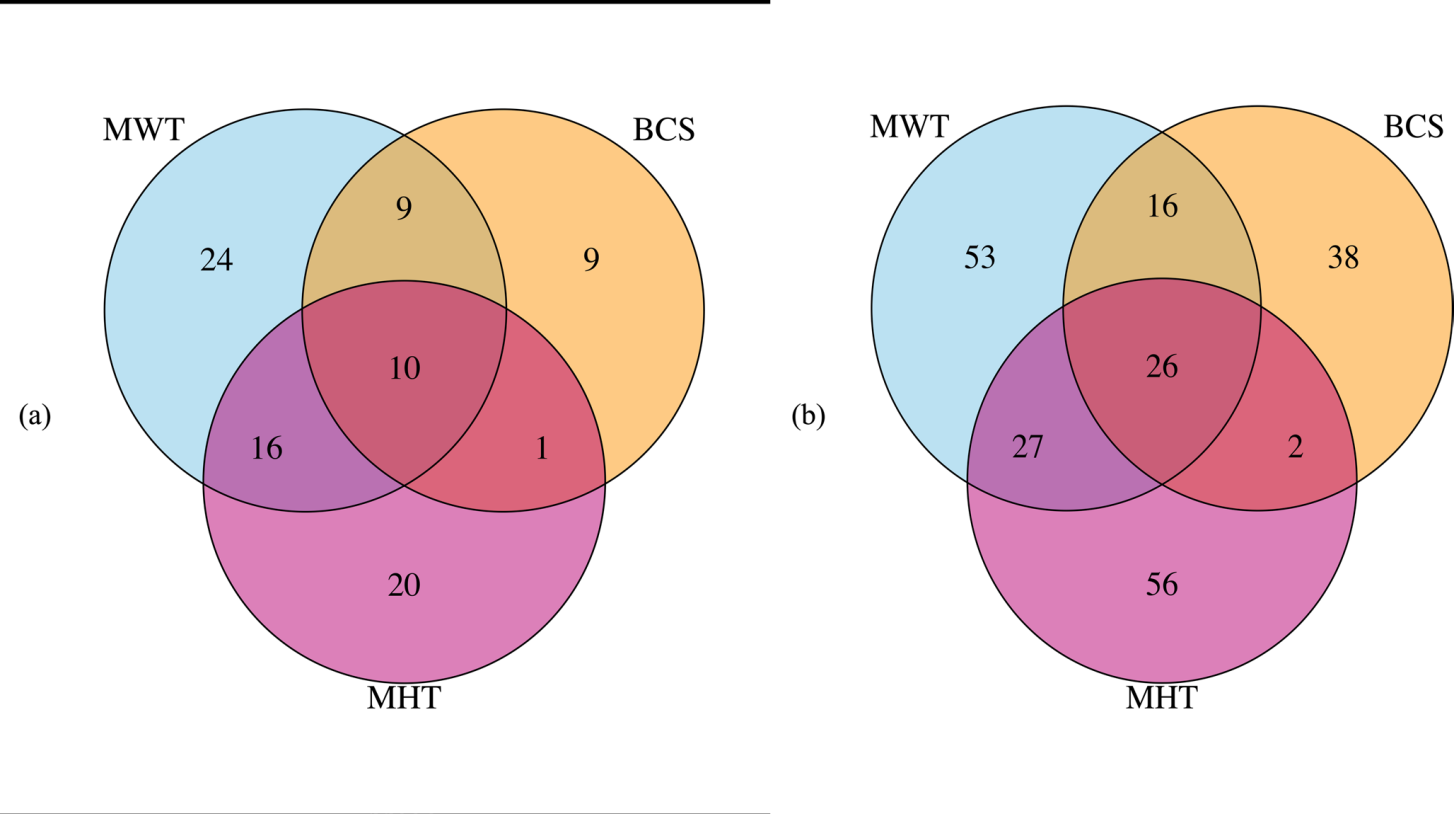
Venn diagram of the number of **a** significant SNPs and **b** genes between mature cow weight (MWT), mature cow height (MHT), and body condition score (BCS) in American Angus cattle population

Figure 2 shows Venn diagrams illustrating the number of significant SNPs and genes associated with mature cow size traits. A total of 24, 9, and 20 SNPs were unique to MWT, BCS, and MHT, respectively, while 10 SNPs were shared among all three traits.

Additional overlaps included 9 SNPs between MWT and BCS, 16 SNPs between MWT and MHT, and 1 SNP between BCS and MHT. Regarding genes, 53, 38, and 56 genes were unique to MWT, BCS, and MHT, respectively. Twenty-six genes were common to all three traits, while 16, 27, and 2 genes were shared between MWT and BCS, MWT and MHT, and BCS and MHT, respectively. Genes such as *ATP6V0E1*, *BNIP1*, *CREBRF*, *ERGIC1*, *NKX2*−5, and *RPL26L1* were shared between MHT and BCS and common to all three traits. Common to MWT and MHT were *ATAD2*, *CCND2*, *DUSP1*, *FBXO32*, *FGF23*, *FGF6*, *NEURL1B*, *NTAQ1*, *SH3PXD2B*, *STC2*, *TIGAR*, *TRAPPC9*, and many more. Some of the genes common to MWT and BCS include *ARRDC3*, *ENC1*, *EPCIP*, *PAXBP1*, *RAB11FIP2*, *SYNJ1*, and *TIAM1*. We also identified overlapping genes on several chromosomes that were consistently detected across studies. On BTA7, *ARRDC3* was identified, while *CCND2* was detected on BTA5. Multiple genes located on BTA20, including *STC2*, *RPL26L1*, *CREBRF*, *BNIP1*, *NKX2-5*, *ERGIC1*, *ATP6V0E1*, *ENC1*, and *SH3PXD2B*, were found in overlapping regions, and *ZFAT* was identified on BTA14 in regions associated with body size and conformation traits.

### Pleiotropic effects

**Fig. 3:**
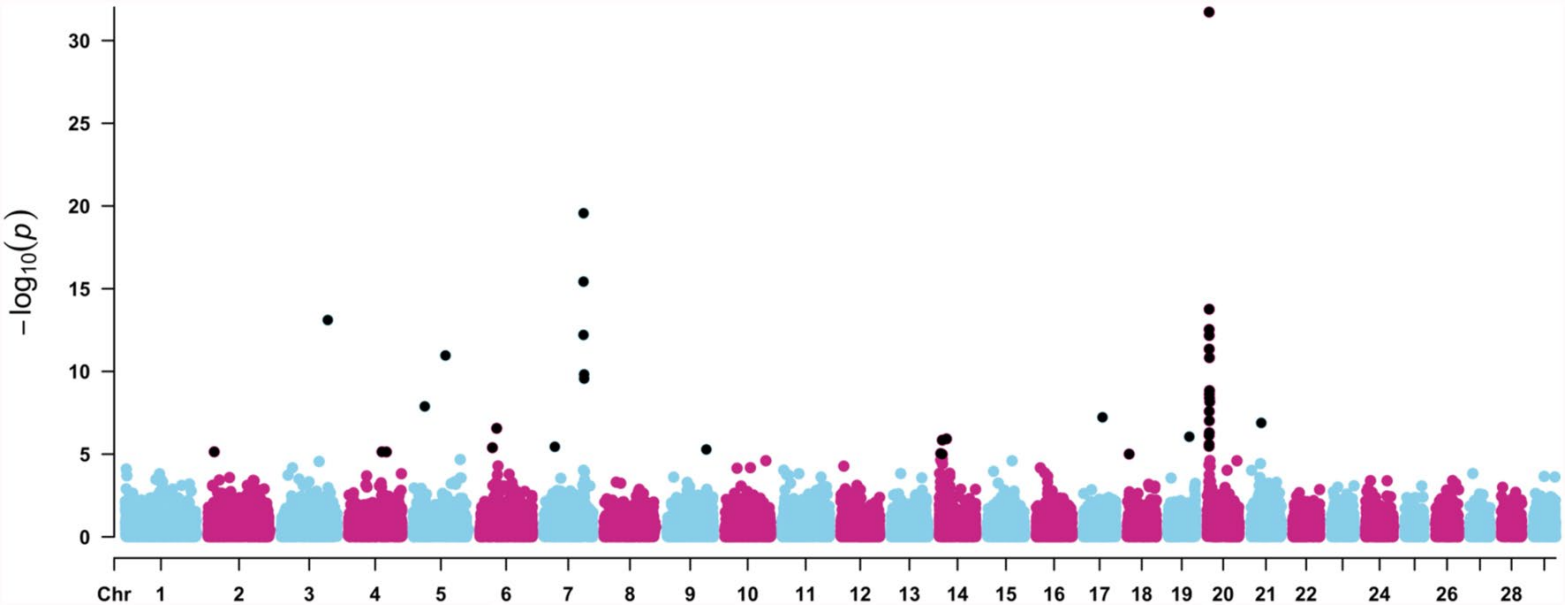
Manhattan plot for multi-trait analysis of mature cow size traits (mature cow weight, height, and body condition score) in American Angus cattle population

Following the method proposed by Bolormaa et al. (2014), we identified 39 significant SNPs with pleiotropic effects across all mature cow size traits (Fig. 3). Within these regions, 81 genes were detected, including 37 protein-coding genes, 37 long non-coding RNAs, 5 microRNAs, 1 ribosomal RNA, and 1 small nucleolar RNA. The genomic regions richest in annotated genes were BTA20, BTA14, and BTA7, with 26, 13, and 9 genes, respectively. Overlap between pleiotropic and single-trait GWAS results included 38 genes for BCS, 48 for MWT, and 54 for MHT, indicating regions consistently detected across approaches. Shared genes between pleiotropy and single-trait analyses were identified for MWT (*ARRDC3*, *ATAD2*, *DUSP1*, *FBXO32*, *NTAQ1*, *STC2*, and *TRAPPC9*), MHT (*ADAMTSL3*, *ATAD2*, *DUSP1*, *FBXO32*, *NKX2-5*, *STC2*, and *TRAPPC9*), and BCS (*AKAP12*, *ARRDC3*, and *MTHFD1L*).

In addition, several genes, including *PECAM1*, *MILR1*, *POLG2*, *CEP95*, and *SMURF2*, were uniquely identified through the pleiotropy analysis, indicating genomic regions detected exclusively by the multi-trait approach. The Manhattan plot for the GWAS of BCS_CT_, MWT_CT_, and MHT_CT_, showing markers significantly associated with each trait individually, is provided in Supplementary Material S4.

### QTL identification and functional analyses

**Fig. 4:**
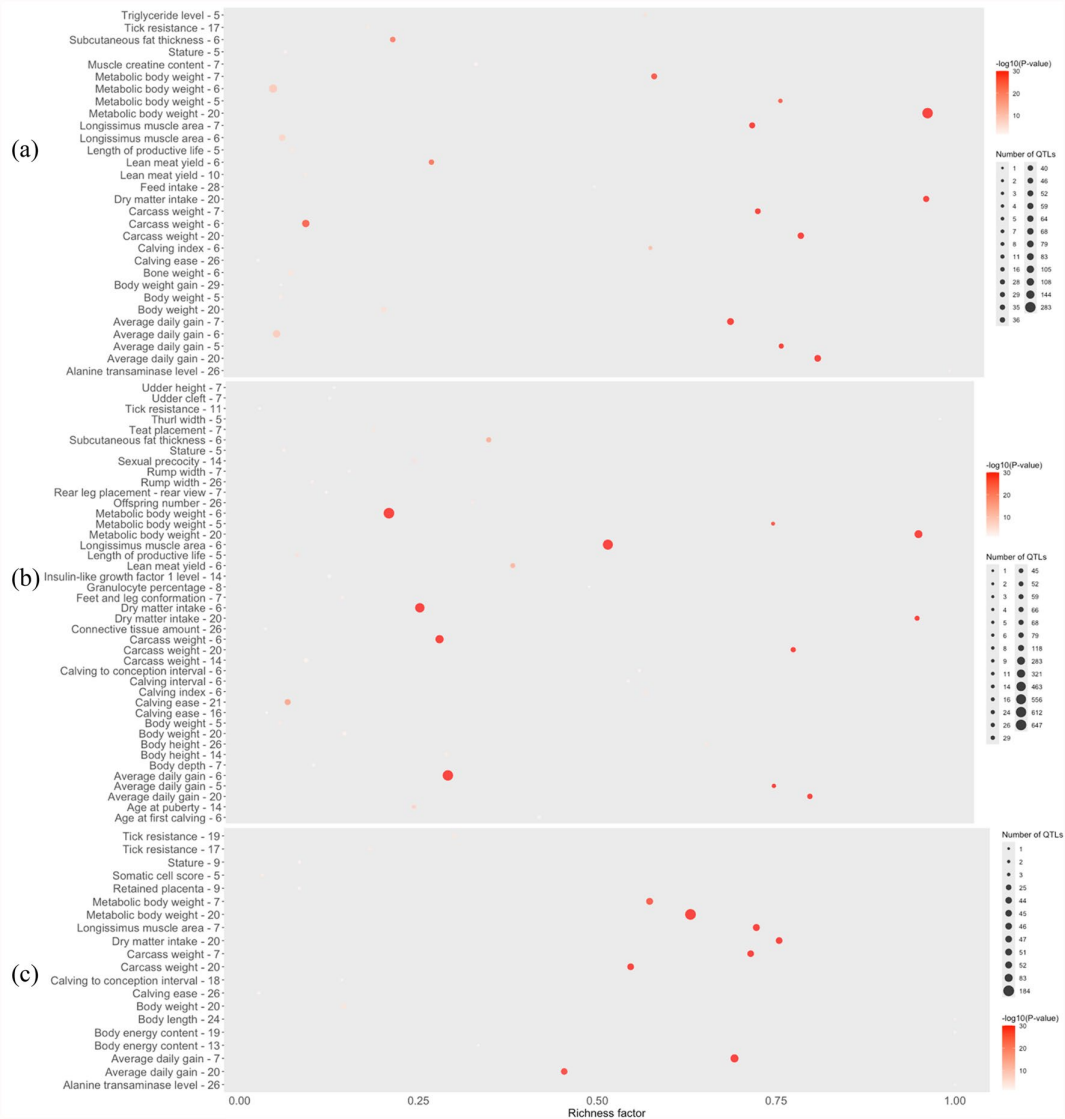
QTL enrichment for the significant regions for **a** mature cow weight, MWT; **b** mature cow height, MHT; and **c** body condition score, BCS, in American Angus cattle population. The y-axis represents QTL categories, the x-axis shows the richness factor (calculated as the number of genes annotated in this study associated with a specific QTL divided by the total number of genes within that QTL), bubble area corresponds to the number of associated QTLs, and bubble color indicates the *p*-value (darker color denotes higher significance)

QTL enrichment for MWT revealed 33 QTLs associated with major trait categories, including production (e.g., body weight, average daily gain, metabolic body weight, dry matter intake, and length of productive life), meat and carcass traits (e.g., bone weight, carcass weight, lean meat yield, longissimus muscle area, muscle creatine content, and subcutaneous fat thickness), health (e.g., triglyceride level and tick resistance), milk production (e.g., milk iron content, milk fat percentage, milk C14, C16, and C18 indices, and several fatty acid components), exterior (stature), and reproduction (e.g., calving ease and calving index; Fig. 4a).

For MHT, QTL enrichment identified 43 QTL distributed across multiple chromosomes. These QTL were assigned to major trait categories, including production, meat and carcass, milk, reproduction, exterior, and health. Notably, several annotations were uniquely related to skeletal growth and body frame, such as stature, body height, body depth, thurl width, rump width, feet and leg conformation, and rear leg placement (Fig. 4b).

Significant associations with BCS were identified across several chromosomes, spanning the major trait categories. A total of 20 QTLs were directly related to growth and body composition, particularly carcass weight, *longissimus* muscle area, body energy content, body length, and average daily gain (Fig. 4c).

**Fig. 5:**
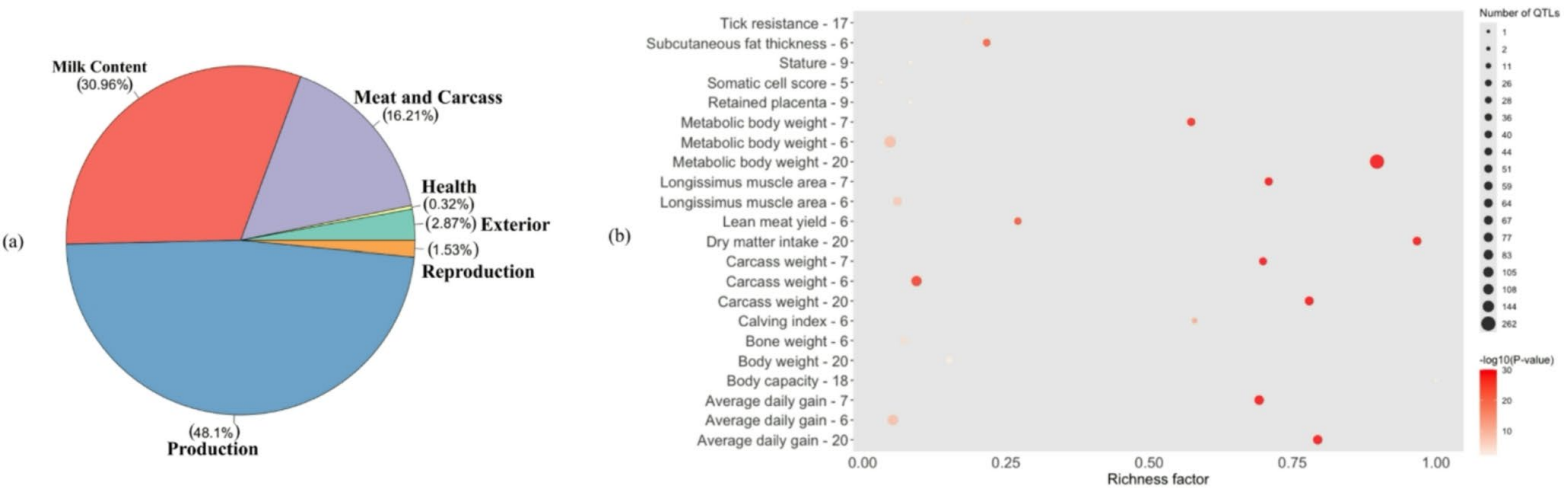
**a** Chart of the trait-types associated with the quantitative trait loci (QTL) overlapping with the pleiotropic genomic regions associated with all mature cow size traits. **b** QTL enrichment for the significant pleiotropic regions associated with all mature cow size traits from multi-trait analysis. The y-axis represents QTL categories, the x-axis shows the richness factor (calculated as the number of genes annotated in this study associated with a specific QTL divided by the total number of genes within that QTL), bubble area corresponds to the number of associated QTLs, and bubble color indicates the *p*-value (darker color denotes higher significance)

QTL enrichment for pleiotropic effects on mature cow size traits revealed 25 QTLs distributed across eight chromosomes. Figure 5 shows the distribution of these QTLs by previously associated trait-type and their richness factor. Of these, 48.1% QTLs were previously associated with production traits, 30.96% with milk content, 16.21% with meat and carcass traits, 2.87% with exterior, 1.53% with reproduction, and 0.32% with health-related traits (Fig. 5a). Traits such as metabolic body weight, dry matter intake, average daily gain, and carcass weight exhibited strong statistical significance (darker red circles), high richness, and many associated QTLs, indicating a polygenic architecture with involvement in multiple biological pathways (Fig. 5b). Highly significant associations were detected on BTA6, BTA7, and BTA20 (e.g., ADG on BTA7, *p* = 1.16 × 10⁻⁵⁸; MBW on BTA20, *p* = 8.39 × 10⁻²⁸⁰), with carcass weight QTLs overlapping MWT and BCS on BTA7 (*p* = 4.61 × 10⁻³¹) and BTA20 (*p* = 2.87 × 10⁻⁵⁸).

**Table 1:** Gene ontology terms for the positional candidate genes annotated for mature cow weight, MWT, mature cow height, MHT, body condition score, BCS and multi-trait association analysis in American Angus cattle population

Trait	Functional Term	Name	Genes
*ˆ*MWT	REAC: R-BTA-74,752	Signaling by Insulin receptor	*ATP6V0E1*, *FGF6*, *FGF23*
	REAC: R-BTA-5,654,720	PI-3 K cascade: FGFR4	*FGF6*, *FGF23*
	REAC: R-BTA-5,654,695	PI-3 K cascade: FGFR2	*FGF6*, *FGF23*
	REAC: R-BTA-5,654,689	PI-3 K cascade: FGFR1	*FGF6*, *FGF23*
	REAC: R-BTA-5,654,228	Phospholipase C-mediated cascade; FGFR4	*FGF6*, *FGF23*
MHT	KEGG:05206	MicroRNAs in cancer	*bta-mir-30d*,* bta-mir-30b*,* CCND2*,* HNRNPK*, *TP623*
	REAC: R-BTA-5,654,219	Phospholipase C-mediated cascade: FGFR1	*FGF6*, *FGF23*
	REAC: R-BTA-5,654,699	SHC-mediated cascade: FGFR2	*FGF6*, *FGF23*
	REAC: R-BTA-5,654,221	Phospholipase C-mediated cascade; FGFR2	*FGF6*, *FGF23*
	REAC: R-BTA-190,322	FGFR4 ligand binding and activation	*FGF6*, *FGF23*
BCS	REAC: R-BTA-9,013,405	RHOD GTPase cycle	*ARHGAP39*, *AKAP12*, *FILIP1*
All	WP: WP1049	G protein signaling pathways	*AKAP12*, *PDE1A*

KEGG: Kyoto encyclopedia of genes and Genomes; REAC: Reactome pathways, WP: Wikipathways

In this study, we identified 30 pathways related to MWT, 39 related to MHT, one related to BCS, and one related to regions with pleiotropic effects. The pathways associated with MWT were primarily involved in growth factor and energy metabolism signaling, including insulin receptor signaling, phosphoinositide 3-kinase (PI3K) and SRC homology 2 domain containing (SHC)-mediated fibroblast growth factor receptor (FGFR) cascades, downstream FGFR signaling, RAF/mitogen-activated protein kinase (MAPK) pathways, and insulin receptor substrate (IRS)-mediated signaling. Pathways associated with MHT included phospholipase C-mediated FGFR cascades, SHC-mediated FGFR signaling, PI3K cascades, insulin receptor signaling, and microRNAs in cancer. The single BCS-associated pathway involved the RHOD GTPase cycle, and the only pathway associated with regions showing pleiotropic effects was G protein signaling pathways. Table 1 shows the gene ontology terms for the positional candidate genes annotated for mature cow size traits.

**Fig. 6:**
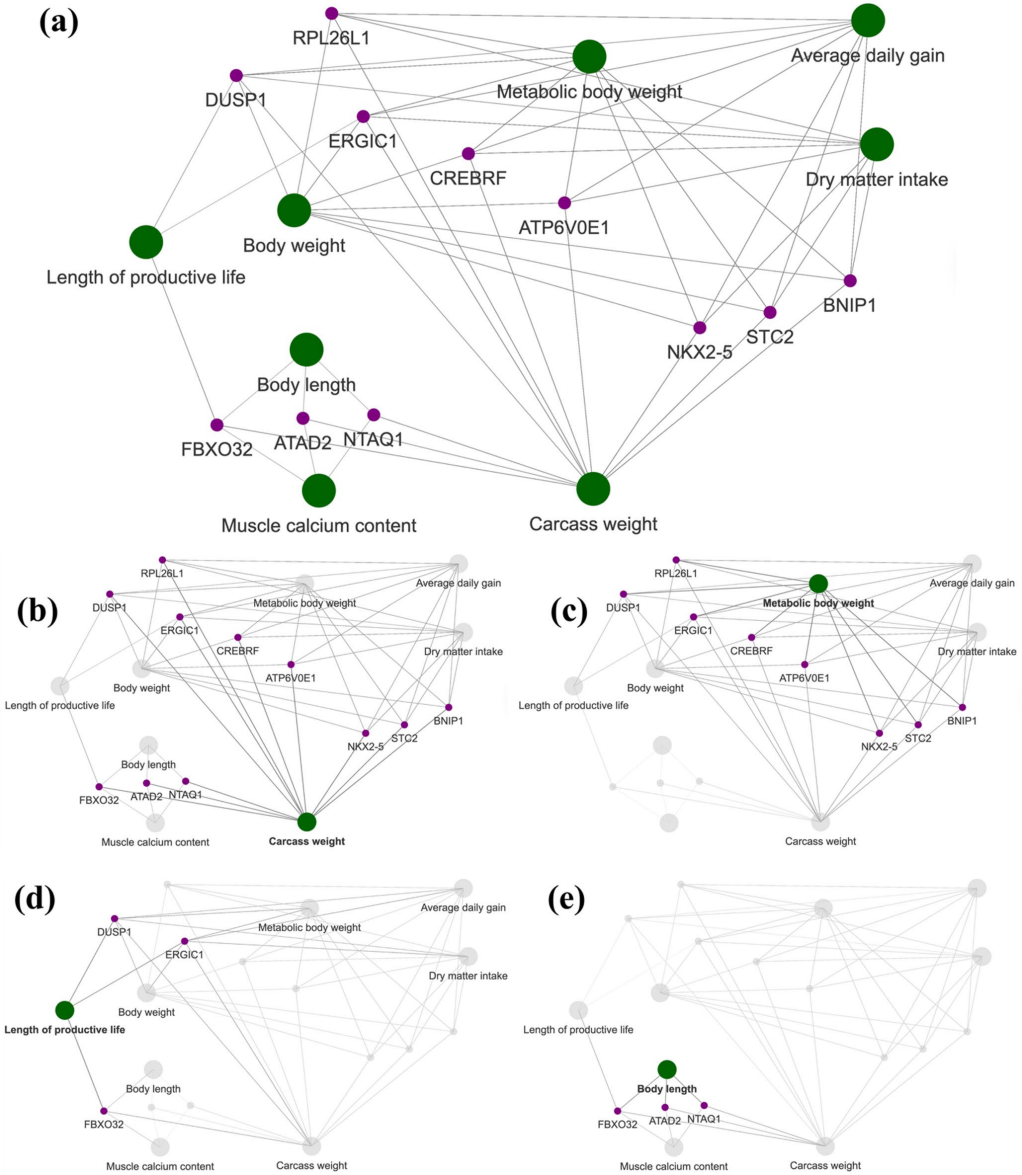
Interaction network composed of quantitative trait loci (QTL) and pleiotropic candidate genes associated with mature cow size traits. **a** Networks showing the relationship between the candidate genes (purple) and the different QTL terms (green); Network highlighting the direct connection between **b** all genes and carcass weight; **c** genes and production-related QTL (metabolic body weight); **d** length of productive life and associated genes; **e** genes and body length

After comparing our GWAS results with the meta-analysis by Bouwman et al. (2018), we identified several candidate genes, including *FBXO32*, *NTAQ1*, *ATAD2*, *DUSP1*, *ERGIC1*, *RPL26L1*, *ATP6V0E1*, *CREBRF*, *BNIP1*, *NKX2-5*, and *STC2*. These genes were associated with QTLs from two major trait categories: meat and carcass (e.g., carcass weight, muscle calcium content) and production (e.g., average daily gain, body weight, dry matter intake, length of productive life, metabolic body weight). A gene–trait interaction network was constructed to visualize the relationships between candidate genes and their corresponding QTLs (Fig. 6). The network revealed two major clusters corresponding to the meat and carcass (Fig. 6b) and production trait categories (Fig. 6c). Within the production cluster, genes such as *DUSP1*, *ERGIC1*, *CREBRF*, *ATP6V0E1*, *STC2*, *NKX2-5*, *BNIP1*, and *RPL26L1* were highly interconnected with traits including body weight, metabolic body weight, average daily gain, and dry matter intake. Conversely, the meat and carcass cluster was dominated by *FBXO32*, *NTAQ1*, and *ATAD2*, which were associated with carcass weight and muscle calcium content. 

Although predominantly associated with meat and carcass traits, these three genes were also associated with body length a production trait (Fig. 6d). Importantly, carcass weight emerged as a central hub linking all identified genes across both clusters. Beyond this, length of productive life also served as a secondary cross-link, connecting *FBXO32* (from the meat and carcass cluster), with *DUSP1*, and *ERGIC1* (from the production cluster), thereby bridging muscle-associated and metabolic traits (Fig. 6e).

## Discussion

Mature cow size traits are economically important in beef cattle production systems and have therefore attracted significant attention in breeding programs. In this study, MWT, MHT, and BCS were investigated through GWAS to characterize their genetic architecture in American Angus cattle. These traits are closely related to feed intake (Lewis And Emmans 2020), maintenance costs (Jenkins And Ferrell 1994; David Lalman 2019), and reproductive efficiency (Pardo et al. 2020), making them central to long-term herd sustainability. The identification of significant genomic regions, candidate genes, and enriched biological pathways provides valuable insights into the genetic mechanisms underlying mature cow size.

### Genomic regions, and biological pathways underlying mature cow size traits

The differences in effective number of independent chromosomal segments and associated significance thresholds across chromosomes reflect differences in marker density and linkage disequilibrium structure, emphasizing the importance of correction factors in GWAS analyses. Significant associations for mature cow size traits were mainly detected on BTA7, BTA14, and BTA20. Among the ten SNPs shared across all traits, three were located on BTA7, and seven on BTA20. Furthermore, several overlapping genes were identified on these key chromosomes, consistent with previously reported associations with growth and body composition traits. Similar to our study, *ARRDC3* was reported on BTA7 (Zhang et al. 2020; Smith et al. 2022; Baneh et al. 2025) to be associated with average daily gain, carcass weight, rib-eye area, and marbling score, while *PENK* was reported on BTA14 (Wang et al. 2020; Smith et al. 2022) to be related to growth traits such as birth weight, weaning weight, and yearling and lean meat yield. In addition, ZFAT on BTA14 was consistently identified by Purfield et al. (2019) within QTL regions related to body size and conformation. Multiple genes located on BTA20, including *STC2*, *RPL26L1*, *CREBRF*, *BNIP1*, *NKX2-5*, *ERGIC1*, *ATP6V0E1*, *ENC1*, and *SH3PXD2B*, were found in similar regions across several studies (Seabury et al. 2017; Zhang et al. 2020; Smith et al. 2022; Baneh et al. 2025). The recurrence of these genes across independent studies supports the robustness of the genomic regions influencing mature cow size.

Consistent associations with yearling weight in Red Angus cattle on BTA7 and BTA20 (Smith et al. 2022) further suggest that these loci contribute to growth trajectories extending from early development through maturity. Likewise, QTLs for carcass weight, rib eye area, and backfat thickness have been mapped to these same regions (Wang et al. 2020; Baneh et al. 2025), emphasizing their relevance to carcass composition and maintenance efficiency. Associations for stature-related traits on BTA7 and BTA14 (Pryce et al. 2011) and the presence of orthologous genes such as *PLAG1*, *CHCHD7*, *RDHE2*, and *NCAPG*, which are also linked to human height, highlight the conservation of growth-regulating pathways across species. Similarly, associations with marbling score detected on BTA7 (Baneh et al. 2025) reflect the close relationship between fat deposition, carcass composition, and variation in BCS.

Mature cow size traits showed extensive overlap with QTLs related to growth, carcass, metabolic body weight, milk, reproduction, and health traits, underscoring the pleiotropic nature of loci influencing size in Angus cattle. The strongest enrichments were observed for average daily gain, metabolic body weight, dry matter intake, and carcass weight, particularly on BTA6, BTA7, and BTA20. These overlaps suggest that loci regulating muscle deposition, fat cover, and feed efficiency also contribute to long-term cow size (Saatchi et al. 2014a; Seabury et al. 2017). Mature cow size was also associated with reproductive QTLs for calving ease, calving index, and calving-to-conception interval. Together, these findings indicate that mature cow size reflects a balance between growth efficiency, maintenance requirements, and reproductive performance (Snelling et al. 2019; Lalman And Beck 2019; Freetly et al. 2023).

Pathway enrichment analyses revealed that MWT was primarily influenced by growth and metabolic pathways, notably the insulin receptor signaling and IRS-mediated PI3K/AKT cascade, which regulate glucose uptake, protein synthesis, and lipid metabolism (Saltiel And Kahn 2001; Copps And White 2012; Boucher et al. 2014). The FGFR signaling and its downstream PI3K/AKT, RAF/MAPK, and PLCγ pathways were also enriched, reflecting their central roles in skeletal growth, muscle differentiation, and adipose regulation (Shaul And Seger 2007; Vanhaesebroeck et al. 2012; Ornitz And Itoh 2015; Berridge And Robinson 2016). Similarly, MHT enrichment underscored FGFR and insulin/IGF1R signaling in bone growth and chondrocyte proliferation (Shaul And Seger 2007; Copps And White 2012; Boucher et al. 2014; Ornitz And Itoh 2015), while microRNA activity suggested post-transcriptional regulation of skeletal genes (McDaneld et al. 2009). For BCS, enrichment of the RHOD GTPase cycle indicated a role in cytoskeletal remodeling, lipid storage, and energy metabolism (Ridley 2006). Across traits, pleiotropy analysis revealed G protein signaling pathways that regulate lipid mobilization, appetite, and energy expenditure (Neves et al. 2002; Etienne-Manneville And Hall 2002; Offermanns 2003), suggesting shared molecular mechanisms controlling growth, energy balance, and body reserves in Angus cattle. Collectively, these findings indicate that mature cow size is influenced by an interconnected network of genes linked to growth, carcass, and fat deposition traits, with BTA7 and BTA20 emerging as major genomic hotspots for selection in Angus populations.

### Candidate genes exclusively associated with mature cow weight

Several genes identified across the associated genomic regions are potential candidates influencing MWT in American Angus cattle. For example, the *HAUS4* gene, previously annotated for effects on cellular morphology, influences tissue-level architecture and contributes to variation in residual feed intake and carcass traits such as rib-eye area (Wang et al. 2020; Zhang et al. 2020). Changes in cell morphology within muscle and adipose tissues could alter tissue structure, thereby affecting carcass composition, feed efficiency, and overall cow size (Cantalapiedra-Hijar et al. 2018; Li et al. 2021). The *MMP14* gene belongs to a group of enzymes called matrix metalloproteinases, which regulate multiple biological processes, including the development and remodeling of muscle, adipose, and connective tissues (Christensen And Purslow 2016; Jeong et al. 2017; Peck et al. 2022). As a membrane-anchored metalloproteinase, *MMP14* contributes to extracellular matrix turnover and collagen remodeling, processes that influence tissue growth, structural integrity, and nutrient partitioning (Pach et al. 2021). Because MWT reflects the cumulative balance of lean and adipose tissue deposition, variation in extracellular matrix remodeling and tissue expansion mediated by *MMP14* may indirectly affect body composition and, consequently, mature cow size.

Additionally, the *NBEA* gene has been reported to play roles in body weight regulation, feed intake, and thermal stress response in cattle (Howard et al. 2014). It has also been identified as a candidate gene for high-altitude acclimation in cattle, yaks, and sheep (Yang et al. 2016; Qi et al. 2019; Edea et al. 2019), suggesting its involvement in metabolic and physiological adaptation under environmental stress. Because MWT reflects long-term energy balance, nutrient utilization, and thermoregulatory efficiency, the association of *NBEA* with both feed intake and temperature regulation indicates that it may influence mature cow size through pathways governing energy metabolism and maintenance requirements (Igoshin et al. 2019). The *ZFAT* gene encodes a zinc finger and AT-hook transcription factor involved in cell survival, immune regulation, and growth processes (Tsunoda et al. 2010). In cattle, a strong association for carcass weight was detected near *ZFAT* on BTA14, and this region has also been linked to stature, feed intake, and a lethal haplotype increasing body weight in Angus populations (Pryce et al. 2011; Purfield et al. 2019). Given its repeated association with body size and weight-related traits, *ZFAT* likely contributes to variation in MWT by influencing skeletal growth and overall frame size (Jenko et al. 2019). Other important genes associated with MWT were found on BTA14, a region known for its association with milk production and composition (Georges et al. 1995; Coppieters et al. 1998; Grisart et al. 2004; Lu et al. 2020). The presence of these genes, including *FAM83H*,* PTK2*,* ZNF623*, and *TRAPPC9*, may reflect shared pathways underlying both growth and lactation traits, such as energy metabolism and developmental regulation (Khan et al. 2022; Tang et al. 2024).

### Candidate genes exclusively linked with mature cow height

Various genes were identified as being exclusively associated with MHT, suggesting their specific involvement in pathways governing skeletal development, bone growth, and overall body frame in Angus cattle. Among these, the *UROS* gene encodes uroporphyrinogen III synthase, an enzyme essential for heme biosynthesis and normal red blood cell function (Clavero et al. 2010; Di Pierro et al. 2016). Deficiency or reduced activity of this enzyme can impair oxygen transport and tissue metabolism, potentially affecting growth and skeletal development, thereby linking *UROS* to variation in MHT through its influence on metabolic efficiency and bone formation (Agerholm et al. 2012; Craig et al. 2016). The *WDPCP* gene plays an essential role in ciliogenesis (the process of assembling a cilium, a hair-like organelle on the surface of a cell, from the mother centriole) and cell polarity, processes that influence cell signaling, tissue organization, and skeletal morphogenesis (Cui et al. 2013; Langhans et al. 2021). In cattle, associations of *WDPCP* with body conformation (Ogunbawo et al. 2025) and tissue integrity (De Las Heras-Saldana et al. 2019; Afonso et al. 2020) indicate that it may affect MHT by regulating coordinated cell growth and differentiation required for normal skeletal elongation. Its involvement in limb and skeletal development suggests that *WDPCP* contributes to variation in structural growth traits such as stature.

Furthermore, the *DEPTOR* gene, identified not only in cattle but also in studies on growth and muscle development in chickens and geese (Volkova et al. 2024; Liu et al. 2025), plays a key role in regulating skeletal muscle satellite cells and skeletal growth pathways, highlighting its potential importance in influencing MHT through effects on muscle and bone development (Perez-Tejeiro And Csukasi 2021). The *LAP3* gene, a member of the lamina-associated polypeptide family involved in cell maintenance and growth, has been associated with body weight in sheep (La et al. 2019), body measurement traits in cattle (An et al. 2019), feed efficiency (Lindholm-Perry et al. 2011; Zhang et al. 2016), and bone weight (Xia et al. 2017; Miao et al. 2018; Chang et al. 2018; Niu et al. 2021), highlighting its potential role in regulating MHT through effects on skeletal growth and body size. Similarly, *MED28*, which functions in cell proliferation and cell cycle regulation (Cho et al. 2019) and has been associated with body weight, intramuscular fat content, and yearling weight in cattle (Santiago et al. 2017; Anton et al. 2018), may contribute to MHT by influencing growth and tissue development. *PENK* (Proenkephalin), through its role in growth regulation and GnRH signaling, has been linked to early growth traits (Utsunomiya et al. 2013; Naserkheil et al. 2020), stature (Lettre et al. 2008; Karim et al. 2011), carcass traits (Seabury et al. 2017; Hay And Roberts 2018; Wang et al. 2020) in cattle, and adult human height (Pryce et al. 2011), supporting its potential relevance to MHT in Angus cattle. Other important genes identified in this study, including *ALPK3*, *FAM184B*, *ASB2*, *TP63*, and *USH2A*, have known biological functions related to growth and height, further supporting their potential roles in regulating stature and height in American Angus cattle (Wang et al. 2020; Smith et al. 2022; Qi et al. 2022; Jang et al. 2025).

### Candidate genes uniquely associated with body condition score

Unique associations with BCS highlight genes that may influence the balance between muscle and fat deposition through cellular and metabolic regulatory mechanisms. For example, the *RECQL4* gene, maintains genomic stability and is involved in cellular processes that underpin growth, muscle and adipose development, and metabolic efficiency (Lyu et al. 2021; Luong And Bernstein 2021). Thus, variation in *RECQL4* activity may influence BCS and mature cow size by affecting energy metabolism, tissue renewal, and overall structural conformation (Picard et al. 2016). The *NEBL* gene encodes an actin-binding protein localized to the Z-disc of striated muscle, where it contributes to cytoskeletal organization and myofibril stability (Pappas et al. 2011; Sánchez et al. 2021). Its expression in muscle and metabolic tissues, along with reported co-expression with puberty-related genes, suggests a role in energy metabolism and tissue remodeling (Keogh et al. 2024). In beef cattle, such functions are relevant to BCS, as *NEBL* may influence lean tissue maintenance and fat deposition through regulation of muscle fiber integrity and cellular energy balance.

The *MTHFD1L* gene, plays a key role in mitochondrial folate metabolism and has been associated with weight loss in Italian Large White pigs (Fontanesi et al. 2017; Faggion et al. 2024), carcass length and rib number in Suhuai pigs (Liu et al. 2023), and skeletal development in poultry (Leishman et al. 2025), underscoring its broad involvement in growth, metabolism, and structural traits that are central to variation in mature cow size. Additionally, the *FILIP1* gene, through its role in skeletal muscle cell differentiation, may influence BCS by affecting the balance between muscle growth and energy reserves, thereby linking structural development with metabolic status in cattle (Reimann et al. 2020; Braga et al. 2023). The candidate gene *ARHGAP39* — identified through somatic cell score associations — regulates cell structure and immune signaling, is a candidate gene for mastitis susceptibility in Holsteins (Wang et al. 2015; Pedrosa et al. 2021). Likewise, *RPL8*, with no known direct function with BCS, affects fat-related pathways in cattle (silencing in mammary cells altered lipid-synthesis genes and their promoter variant reduced expression) and thus may influence BCS by impacting fat stores and energy balance (Zheng et al. 2019).

### Candidate genes exhibiting pleiotropic effects

Pleiotropy, the phenomenon where a single genetic locus influences multiple traits, is a primary driver of genetic correlations observed between different complex traits (Solovieff et al. 2013). These genetic correlations arise because the same genetic variation can impact more than one phenotype, leading to interconnectedness in their expression. For instance, high genetic correlations have been reported between mature cow size traits, such as MWT and MHT (Ojo et al. 2025). However, it is important to distinguish true pleiotropy from other phenomena like linkage disequilibrium, where separate but physically close genes on a chromosome are inherited together, or spurious associations that can arise from biases in experimental design or analysis (Gilbert And Le Roy 2007; Bolormaa et al. 2010; Yang et al. 2013). Identifying and characterizing these pleiotropic effects, particularly through multiple-trait GWAS, is crucial for unraveling the complex biological pathways that regulate multiple phenotypes. In this context, multi-trait GWAS offers advantages over single-trait analyses by increasing power to detect QTL, improving mapping precision through correlated traits, and distinguishing true pleiotropy from linked loci, thereby resolving whether associations reflect a single pleiotropic QTL or multiple linked variants and providing clearer insights into the genetic architecture (Knott And Haley 2000; Korol et al. 2001).

In our study, several genes, including *CCND2*, *FGF23*, *FGF6*, *SH3PXD2B*, *TIGAR*, and *TRAPPC9*, were shared between MWT and MHT. The *CCND2* gene has been previously associated with average daily gain, metabolic body weight (Zhang et al. 2020), stature (Bouwman et al. 2018), growth traits and carcass muscle mass in beef cattle (Seabury et al. 2017), and obesity in humans (Elbein et al. 2011). It also plays a key role in the proliferation and differentiation of both pre-adipocytes (Wei et al. 2016) and muscle cells (Zhou et al. 2022). Its involvement in myogenesis and adipogenesis suggests that *CCND2* contributes to variation in morphological traits of animals by influencing muscle development and fat deposition. Similar to *CCND2*, the *FGF6* and *FGF23* genes, which belong to the fibroblast growth factor family, were associated with the rump structure and showed enrichment for pathways involved in cell regulation, differentiation, signaling, and tissue morphogenesis (Silva et al. 2024). These genes are mainly expressed in bone tissue, where *FGF23* contributes to phosphate regulation and vitamin D metabolism—two processes that are vital for bone formation and maintenance (Onal et al. 2018; Imel et al. 2019). Because phosphate plays essential roles in bone mineralization, skeletal development, and cellular signaling, changes in *FGF23* expression may influence both metabolic and structural traits (Blau And Collins 2015). In turn, *FGF6* has been implicated in differences in body size (Ghoreishifar et al. 2020), muscle growth (Fang et al. 2020), and conformation (Bernard et al. 2009; Silva et al. 2024). The combined influence of *FGF6* and *FGF23* on muscle and skeletal development explains why they emerge as candidate genes with pleiotropic effects on both MWT and MHT in Angus cattle. Studies have shown that *NEURL1B* influences the *longissimus dorsi* muscle area in cattle (Li et al. 2017) while *SH3PXD2B* is essential for normal postnatal growth and tissue development (Mao et al. 2009). Together, their involvement in muscle formation and overall growth regulation suggests that variation in these genes may contribute to differences in MWT and MHT.

We also identified a few genes that were peculiar to MWT and BCS, such as *ARRDC3*, *ENC1* and *TIAM1*. The *ARRDC3* gene has been previously associated with carcass traits such as hot carcass weight, rib-eye area, and marbling score (Wang et al. 2020; Baneh et al. 2025), as well as with residual feed intake, dry matter intake, average daily gain, and metabolic body weight in cattle (Zhang et al. 2020). The *ARRDC3* gene regulates obesity in mice and in human males, suggesting a broader role in energy balance and adiposity—both of which are central to overall body size in cattle (Patwari et al. 2011; Patwari And Lee 2012). Likewise, *ENC1*, previously associated with residual feed intake and carcass weight in Angus cattle (Zhang et al. 2020; Baneh et al. 2025), underscores its role in regulating feed efficiency and tissue accretion, processes closely tied to body size and conformation in cattle. The *TIAM1* gene, previously associated with average backfat thickness in cattle (Li et al. 2022), encodes a RAC1-specific guanine nucleotide exchange factor that regulates RAC1-mediated signaling pathways involved in cell morphology, polarity, growth, and survival. Through its influence on actin cytoskeleton organization, membrane trafficking, and cellular adhesion, *TIAM1* contributes to tissue remodeling and lipid metabolism. The identification of *TIAM1* for both MWT and BCS suggests shared molecular pathways underlying muscle growth and fat deposition, consistent with its previously reported role in adipose tissue development and metabolic regulation. Genes detected for MHT and BCS overlapped with those identified across all three traits and closely corresponded to previously identified pleiotropic candidate genes. These identified pleiotropic genes are discussed in detail in the subsequent section.

Unique genes identified in the pleiotropy analysis included *PECAM1*, which is previously linked with dry matter intake (Li et al. 2021), meat quality (Bruscadin et al. 2021), carcass traits (Niu et al. 2021), immune response, and growth rate (de Lima et al. 2020), suggesting a broad role in regulating efficiency, growth and body reserves in Angus cattle. Likewise, Naserkheil et al. (2020) reported *PECAM1*,* MILR1*,* POLG2*,* SMURF2*, and *DDX5* as associated with rib-eye area in Hanwoo beef cattle, and these genes were also detected in our pleiotropy-based analysis, supporting their potential involvement in mature cow size. In addition, the genes *PECAM1*,* MILR1*,* POLG2*, and *DDX5* have been associated with choline metabolism, in relation to dry matter intake, rib-eye area, and marbling score in beef cattle (Li et al. 2021, 2022). Because choline plays a key role in lipid metabolism, muscle development, and energy balance, these genes may contribute to pleiotropic effects on mature cow size traits(Li et al. 2015; Moretti et al. 2020).

Notably, *SMURF2* has been shown in mice to regulate bone remodeling by controlling osteoblast–osteoclast communication and bone mass homeostasis (Xu et al. 2017). Given the importance of skeletal development in determining overall body size, this function provides a strong biological basis for its pleiotropic association with MWT, MHT, and BCS in Angus cattle. The *PECAM1*, *POLG2*, *DDX5*, *CEP95*, and *SMURF2* genes have also been associated with intramuscular fat and fatty acid composition of the *Longissimus thoracis et lumborum* muscle in rabbits (EL Nagar et al. 2023), pointing to their roles in regulating muscle quality and lipid metabolism. In addition, *PECAM1*, *MILR1*, *POLG2*, *SMURF2*, *CEP95*, and *DDX5* were linked to rear limb cannon bone circumference in Yorkshire pigs through a pleiotropy study (Qiu et al. 2024), highlighting their contribution to skeletal growth and structural development. Taken together, these cross-species associations suggest that these genes may influence both compositional (muscle and fat deposition) and structural (skeletal size) components of growth, providing a strong biological basis for their pleiotropic effects on mature cow size traits. We also identified *ADAMTSL3* and *ADAMTS18*, members of the *ADAMTS* gene family known for their diverse biological roles in cell differentiation, adhesion, and tissue remodeling (Seegar And Blacklow 2019). The *ADAMTSL3* gene has previously been reported as a significant genetic marker for body traits in beef cattle and associated with growth traits such as body weight at one year of age in Brahman cattle (Liu et al. 2012; Martínez et al. 2017), and body conformation traits in Nellore cattle (Machado et al. 2022), highlighting its role in growth regulation. Similarly, *ADAMTS18* has been reported to play a role in heat stress response in Indian Sahiwal cattle (Rajawat et al. 2024). Their detection in our analysis suggests that variation in these genes may contribute to pleiotropic effects on mature cow size through pathways regulating growth, structural development, energy balance, and indirectly through adaptation to environmental stressors.

### Pleiotropic candidate genes for mature cow size traits with cross-study evidence

Bouwman et al. (2018) reported that variation in stature across cattle breeds is regulated by a shared set of highly polygenic genes similar to those in humans. This provides an important reference for comparing the pleiotropic candidate genes identified in our study for mature cow size traits. Aside from the genes shared among all three traits and identified in the pleiotropy-based approach (*ATP6V0E1*, *BNIP1*, *CREBRF*, *ERGIC1*, *NKX2-5*, and *RPL26L1*), we also identified additional pleiotropic candidate genes such as *ATAD2*, *DUSP1*, *FBXO32*, *NTAQ1*, and *STC2.* The interconnected network among these pleiotropic genes highlights their coordinated influence on both growth and metabolic processes. Genes such as *CREBRF*, *ATP6V0E1*, *STC2*, *BNIP1*, *NKX2-5*, *ERGIC1*, and *RPL26L1*, which were closely associated with production traits (e.g., metabolic body weight, dry matter intake, and average daily gain), point to a strong metabolic component underlying mature cow size. These genes are primarily involved in energy homeostasis, fat deposition, and cellular metabolism—biological processes that support sustained growth and maintenance efficiency (Schumacher et al. 2022; Rauw et al. 2025). For instance, *CREBRF* and *ATP6V0E1* are central regulators of energy balance, with the former promoting lipid accumulation and the latter contributing to mitochondrial proton transport and feed efficiency (Kong et al. 2016; Minster et al. 2016). Similarly, *BNIP1* and *NKX2-5* participate in endoplasmic reticulum stress responses and cardiac or muscle tissue development, which may influence how energy and nutrients are partitioned between growth and maintenance functions (Reamon-Buettner And Borlak 2010; Udager et al. 2014; Saatchi et al. 2014b). The *STC2* gene has been associated with cell proliferation in several types of cancers, as well as postnatal growth, skeletal muscle and bone development in mice (Gagliardi et al. 2005; Chang et al. 2008; Kita et al. 2011), and fat accumulation and obesity in non-diabetic humans (Sharma et al. 2008). *ERGIC1* and *RPL26L1* are primarily involved in protein transport and translation efficiency, and both genes have been linked to metabolic body weight in cattle (Seabury et al. 2017; De Las Heras-Saldana et al. 2019). Notably, these two genes are located within the same chromosomal region as *STC2*, *NKX2-5*, *BNIP1*, and *CREBRF* (Zhang et al. 2020; Baneh et al. 2025). Their co-localization within this region, coupled with their associations with all mature cow size traits, suggests that these genes exert pleiotropic effects rather than acting through linkage. Moreover, these loci overlap with those identified in a cross-species study of stature in cattle, humans, and dogs (Bouwman et al. 2018), reinforcing their conserved biological role in regulating body size across mammals.

In contrast, genes predominantly associated with muscle calcium content—*FBXO32*, *NTAQ1*, and *ATAD2*—reflect structural and muscle-related processes driving variation in mature size(Bodine et al. 2001; Gomes et al. 2001; Wang et al. 2012a). *FBXO32*, a key regulator of skeletal muscle protein turnover, plays a dual role in promoting muscle accretion during growth and preventing excessive muscle degradation under stress (Wang et al. 2012a). *ATAD2*, known for its role in chromatin remodeling and cell proliferation, may mediate growth rate and muscle fibre differentiation, as demonstrated in differentiation studies of mammalian cells(Koo et al. 2016; Morozumi et al. 2016; Naserkheil et al. 2020). Meanwhile, *NTAQ1* contributes to post-translational protein modification relevant to muscle metabolism (Wang et al. 2009). Collectively, all these pleiotropic candidate genes were associated with carcass weight, which emerged as a central hub connecting the production and meat-related clusters in the gene–QTL network. This shared association underscores the biological interdependence between muscle deposition, energy metabolism, and growth regulation in determining mature cow size. Genes such as *FBXO32*, *ATAD2*, and *NTAQ1*, which influence muscle growth and structural development, likely contribute to carcass weight through their effects on muscle fiber formation and protein turnover. Conversely, genes within the metabolic cluster may impact carcass weight indirectly by modulating feed efficiency, energy utilization, and adipose deposition. The shared connection of these genes through carcass weight in the network suggests that muscle deposition and energy metabolism are not independent processes but jointly shape the phenotypic expression of mature cow size. Furthermore, the link between length of productive life and genes such as *FBXO32*, *DUSP1*, and *ERGIC1* implies that the same biological pathways influencing growth and muscle maintenance also extend to longevity and metabolic resilience. Together, these patterns show that pleiotropy among mature cow size traits arises from an integrated regulation of muscle growth, energy metabolism, and maintenance efficiency, rather than isolated genetic effects on individual phenotypes.

## Conclusion

This study provides a comprehensive characterization of the genetic architecture underlying mature cow size traits in American Angus cattle, including MWT, MHT, and BCS. Our GWAS identified significant genomic regions, candidate genes, and pleiotropic loci—particularly on BTA7, BTA14, and BTA20—confirming their central roles in growth, feed efficiency, skeletal development, muscle deposition, and energy balance. A set of genes, including FBXO*32*, *NTAQ1*, *ATAD2*, *DUSP1*, *ERGIC1*, *RPL26L1*, *ATP6V0E1*, *CREBRF*, *BNIP1*, *NKX2-5*, and *STC2* emerged as candidate genes exhibiting pleiotropic effects across mature cow size traits, consistent with those reported in the meta-analysis of stature used for comparison. Annotation of QTL and functional enrichment revealed overlapping associations with growth, carcass, metabolic, milk, and reproductive traits, demonstrating the interconnected nature of these physiological systems in regulating mature cow size. Overall, these findings not only identify candidate genes and pathways for mature cow size traits but also provide biological insights into their pleiotropic regulation, offering potential targets for genomic selection to improve growth efficiency and structural development in Angus cattle. Future functional studies are needed to validate these loci and elucidate their precise roles in trait variation and energy metabolism.

## Supplementary Information

Below is the link to the electronic supplementary material.

Supplementary Material 1

## Data Availability

No datasets were generated or analysed during the current study.
