# Supplementary material for: Genome-wide association study of mature cow size traits in American Angus cattle
Source: Mamm Genome. 2026 Apr 6;37(1):53. doi: 10.1007/s00335-026-10226-3 (PMC13053394; doi:10.1007/s00335-026-10226-3)
Supplement: Supplementary file 1 — Supplementary Material 1 [file 335_2026_10226_MOESM1_ESM.docx]

**SUPPLEMENTARY MATERIAL**

**
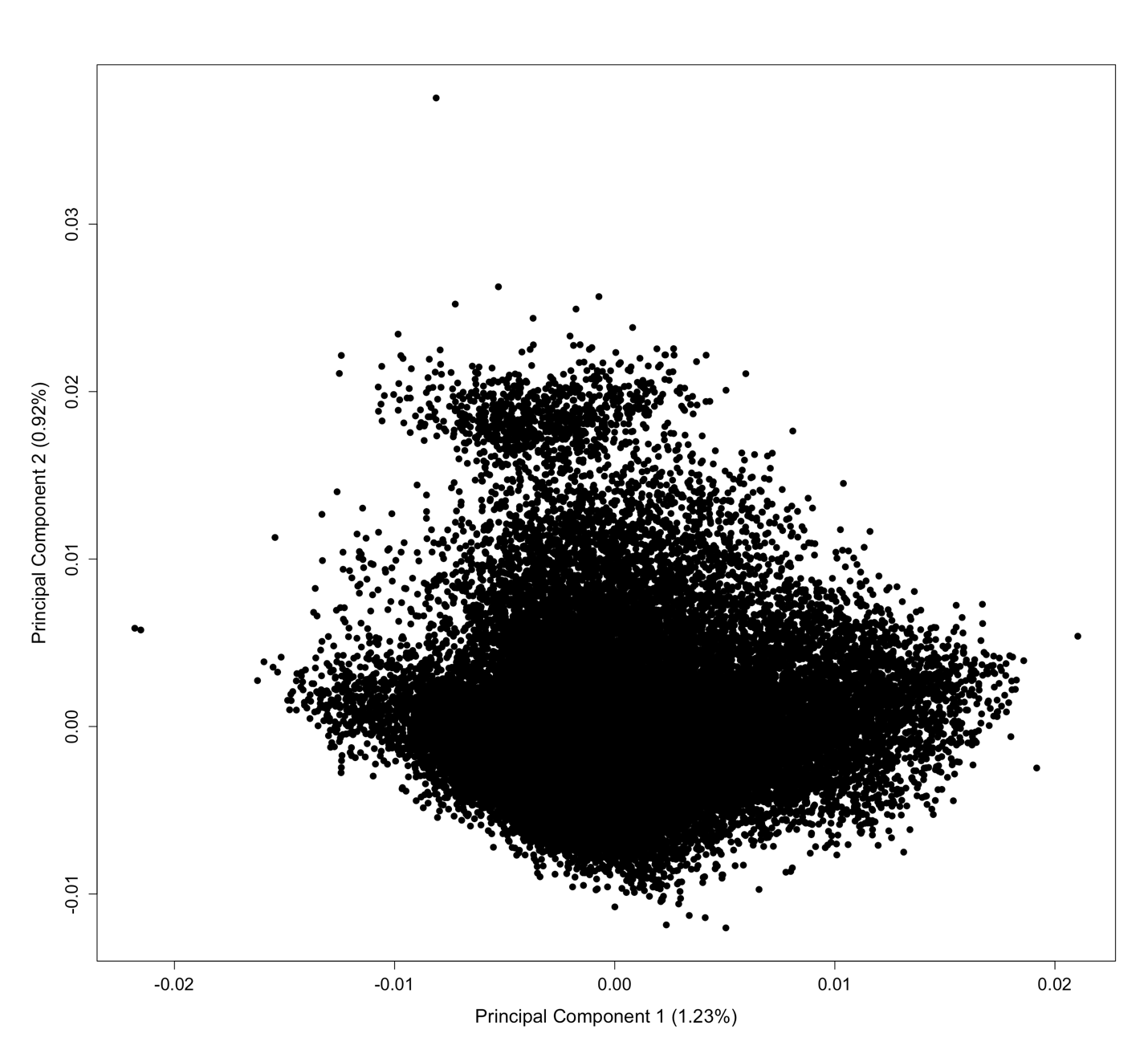
**

**S1.** Principal component (PC) analysis plot of the studied population (PC1 vs PC2)

**
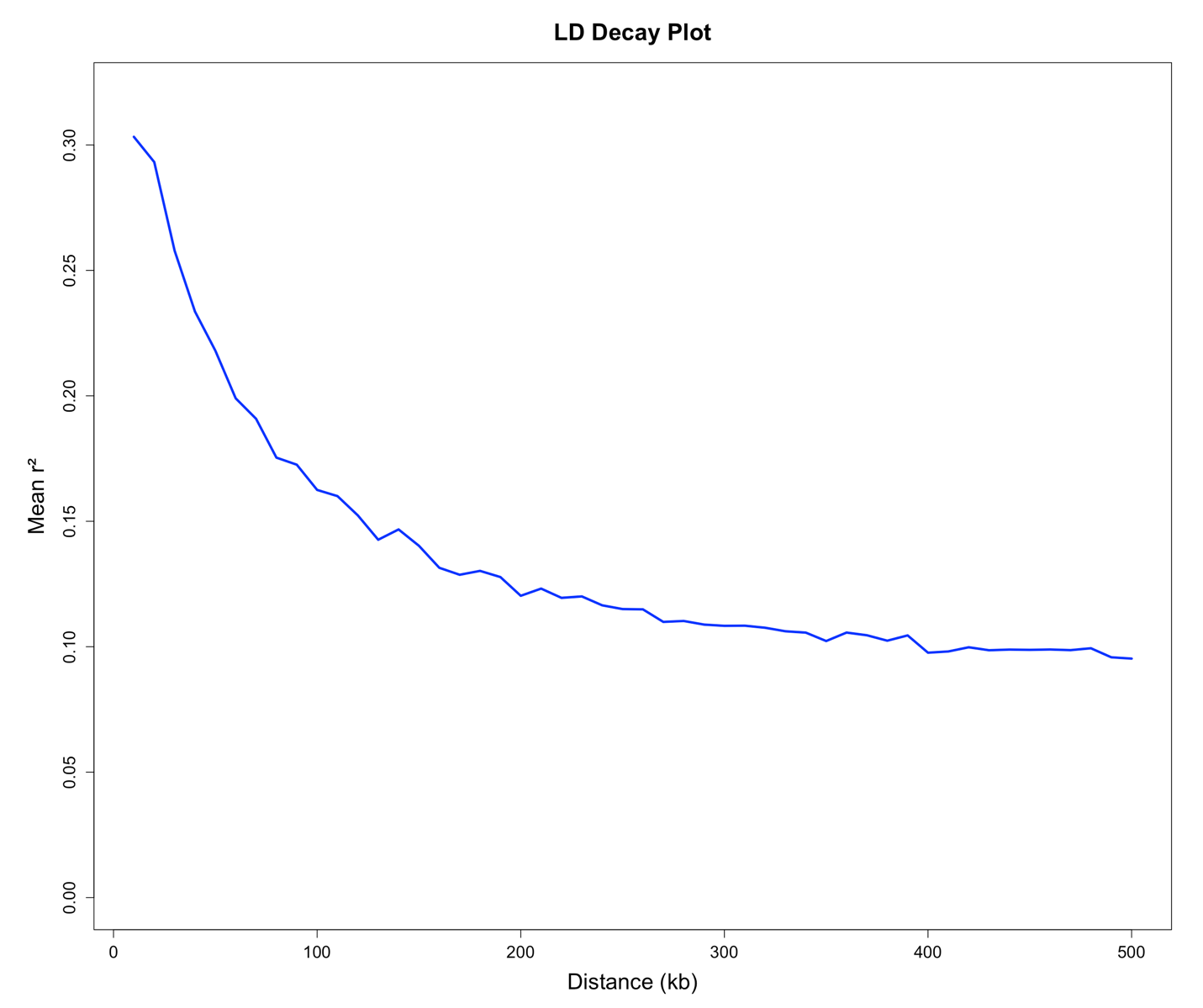
**

**S2.** Linkage disequilibrium (LD) decay based on mean r² (squared correlation between SNP alleles) across physical distance in the studied population.

**S3.** Estimated effective number of independent chromosomal segments (Me), chromosome length (Mbp; megabase pairs), and adjusted chromosome-wide significance thresholds (threshold) for the 29 *Bos* *taurus* autosomes.

| **Chr** | **Length (Mbp)** | **Me*** | **Threshold** |
| --- | --- | --- | --- |
| 1 | 160.25 | 5,673.66 | 5.06 |
| 2 | 138.85 | 4,985.49 | 4.99 |
| 3 | 125.43 | 4,549.25 | 4.96 |
| 4 | 119.65 | 4,360.26 | 4.94 |
| 5 | 124.56 | 4,520.98 | 4.96 |
| 6 | 117.60 | 4,292.80 | 4.93 |
| 7 | 110.14 | 4,047.31 | 4.91 |
| 8 | 115.99 | 4,239.96 | 4.93 |
| 9 | 104.22 | 3,851.04 | 4.89 |
| 10 | 103.11 | 3,814.23 | 4.88 |
| 11 | 106.81 | 3,937.07 | 4.90 |
| 12 | 86.86 | 3,270.06 | 4.82 |
| 13 | 82.94 | 3,137.65 | 4.80 |
| 14 | 81.98 | 3,105.15 | 4.79 |
| 15 | 84.22 | 3,181.03 | 4.80 |
| 16 | 80.57 | 3,057.15 | 4.79 |
| 17 | 72.92 | 2,795.88 | 4.75 |
| 18 | 65.47 | 2,538.98 | 4.71 |
| 19 | 62.98 | 2,452.67 | 4.69 |
| 20 | 71.88 | 2,760.10 | 4.74 |
| 21 | 69.02 | 2,661.78 | 4.73 |
| 22 | 60.53 | 2,367.37 | 4.68 |
| 23 | 52.28 | 2,077.52 | 4.62 |
| 24 | 63.37 | 2,466.07 | 4.69 |
| 25 | 43.31 | 1,757.12 | 4.55 |
| 26 | 51.89 | 2,063.57 | 4.62 |
| 27 | 45.31 | 1,829.03 | 4.56 |
| 28 | 45.59 | 1,838.96 | 4.57 |
| 29 | 50.82 | 2,025.64 | 4.61 |

*****Me was calculated as (Me = $\frac{2\times Ne\times L}{\log(Ne\times L)})$, where Ne is the effective population size (182; Lozada-Soto et al., 2020), and L is the chromosome length in megabase pairs (Mbp). Chromosome-wide significance thresholds were computed as ($threshold= -\log_{10} \frac{0.05}{Me}$). Chr: chromosome**.**


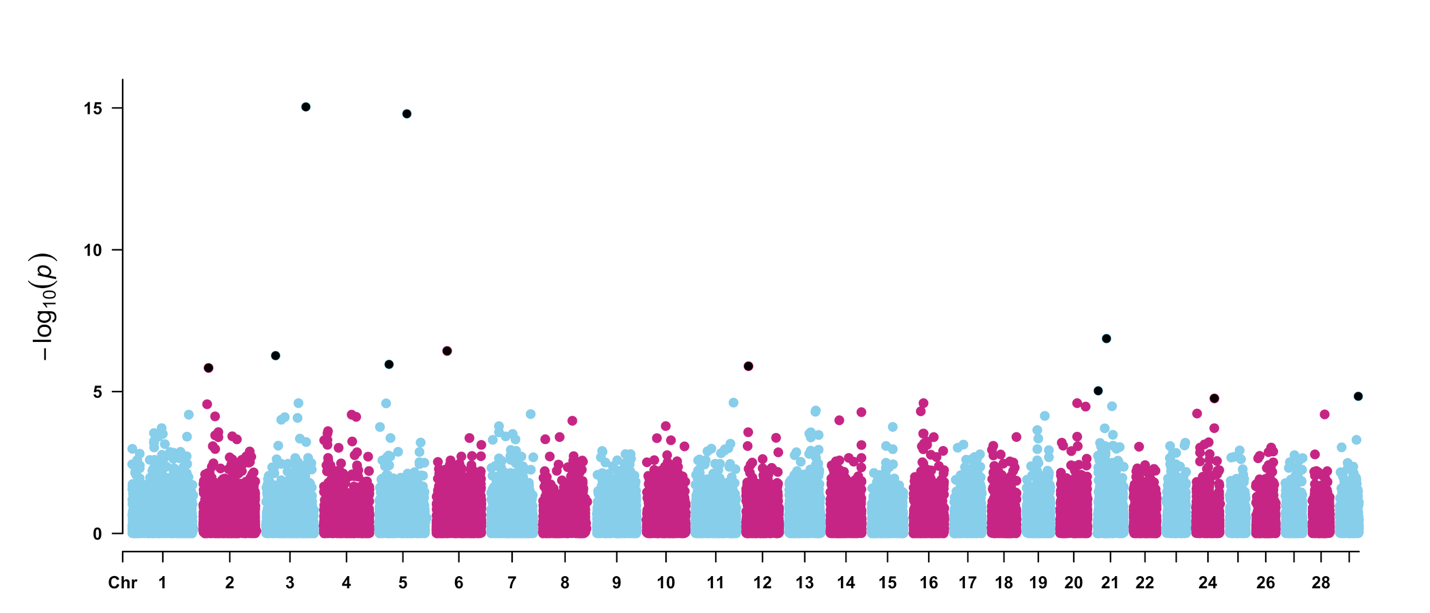

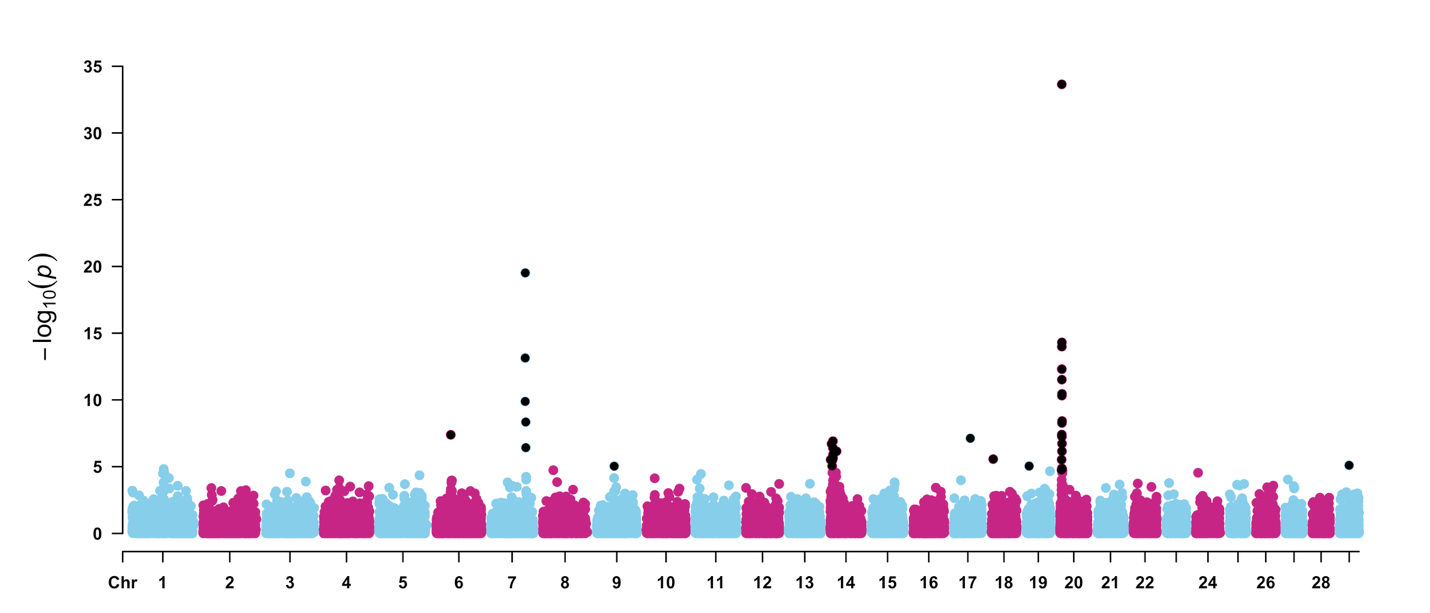

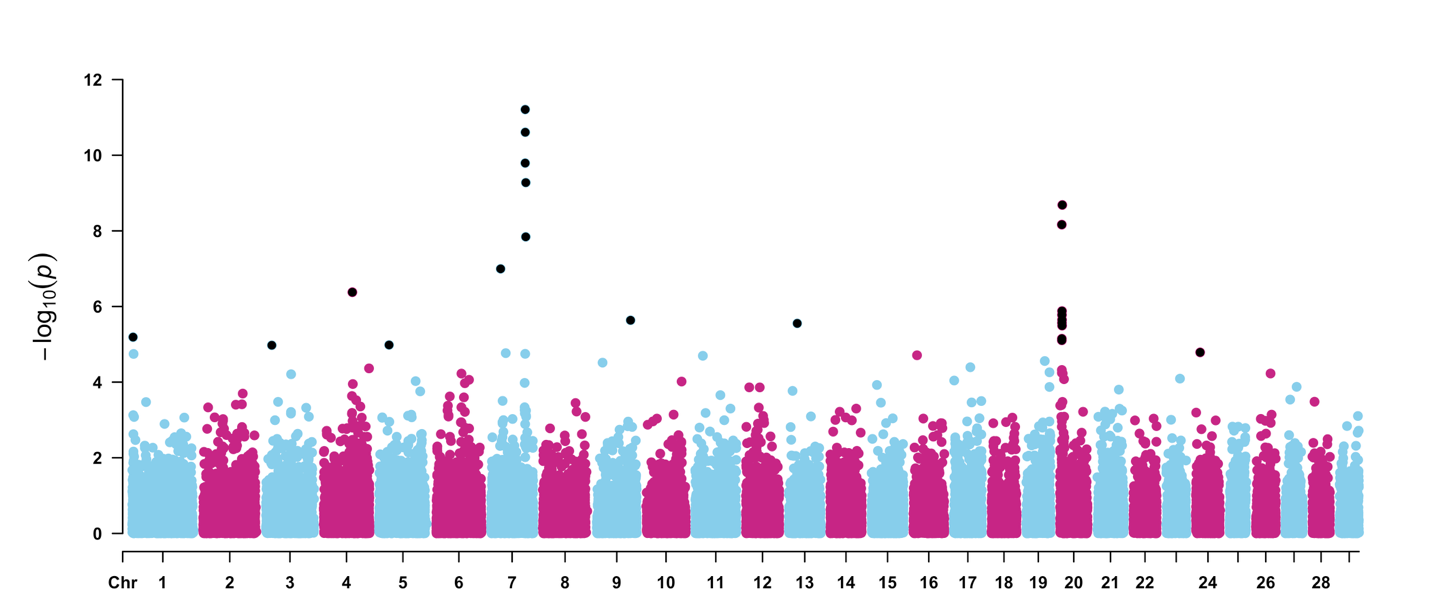


(a)

(b)

(c)

**S4.** Manhattan plot for Cholesky-transformed traits (a) body condition score, BCS_CT_ (b) mature cow weight, MWT_CT_ (c) mature cow height, MHT_CT_, in American Angus cattle population. Black dots represent the significant markers.
